# Supplementary material for: LaMg6Ga6S16: a chemical stable divalent lanthanide chalcogenide
Source: Nat Commun. 2024 Apr 5;15:2959. doi: 10.1038/s41467-024-47209-4 (PMC11271512; doi:10.1038/s41467-024-47209-4)
Supplement: Supplementary file 1 — Supplementary Information [file 41467_2024_47209_MOESM1_ESM.pdf]

Supplementary Information

$LaMg_6Ga_6S_{16}$: A Chemical Stable Divalent Lanthanide Chalcogenide

Yujie Zhang[1], Jiale Chen[1], Kaixuan Li[1], Hongping Wu[1], Zhanggui Hu[1], Jiyang Wang[1], Yicheng Wu[1] and Hongwei Yu[1] ✉

[1]Tianjin Key Laboratory of Functional Crystal Materials, Institute of Functional Crystal, College of Materials Science and Engineering, Tianjin University of Technology, Tianjin 300384, China. ✉email: yuhw@email.tjut.edu.cn

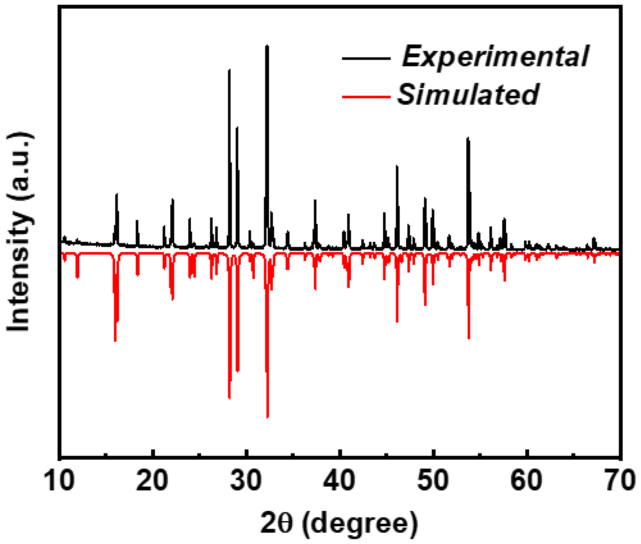

Supplementary Figure 1. Experimental and calculated XRD patterns for $LaMg_6Ga_6S_{16}$.

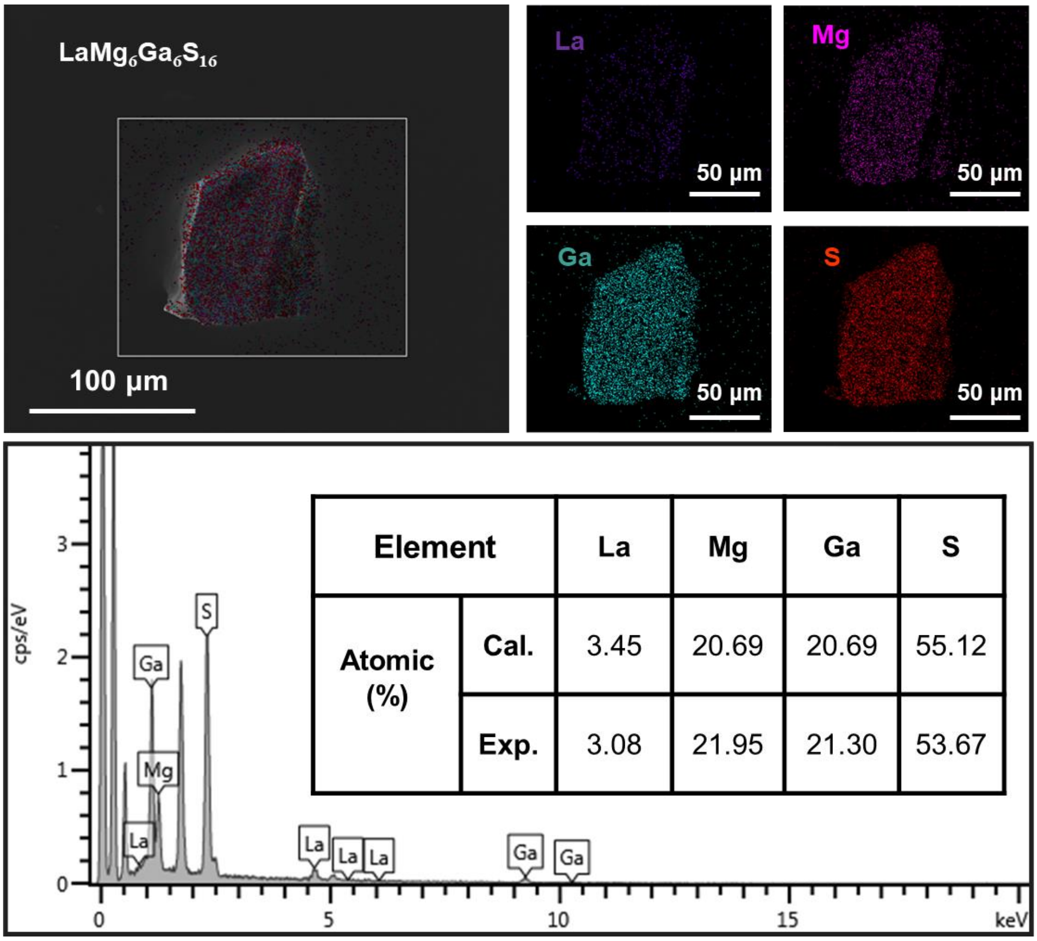

Element		La	Mg	Ga	S
Atomic (%)	Cal.	3.45	20.69	20.69	55.12
	Exp.	3.08	21.95	21.30	53.67

Supplementary Figure 2. The EDS spectra of $LaMg_6Ga_6S_{16}$.

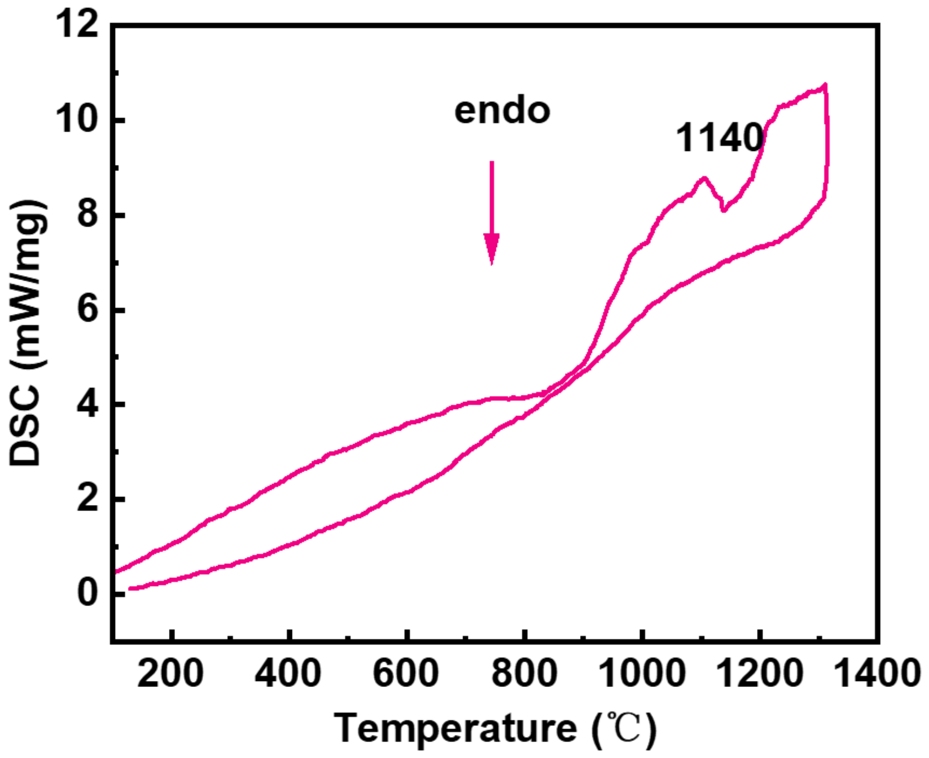

Supplementary Figure 3. DSC curve of $LaMg_6Ga_6S_{16}$.

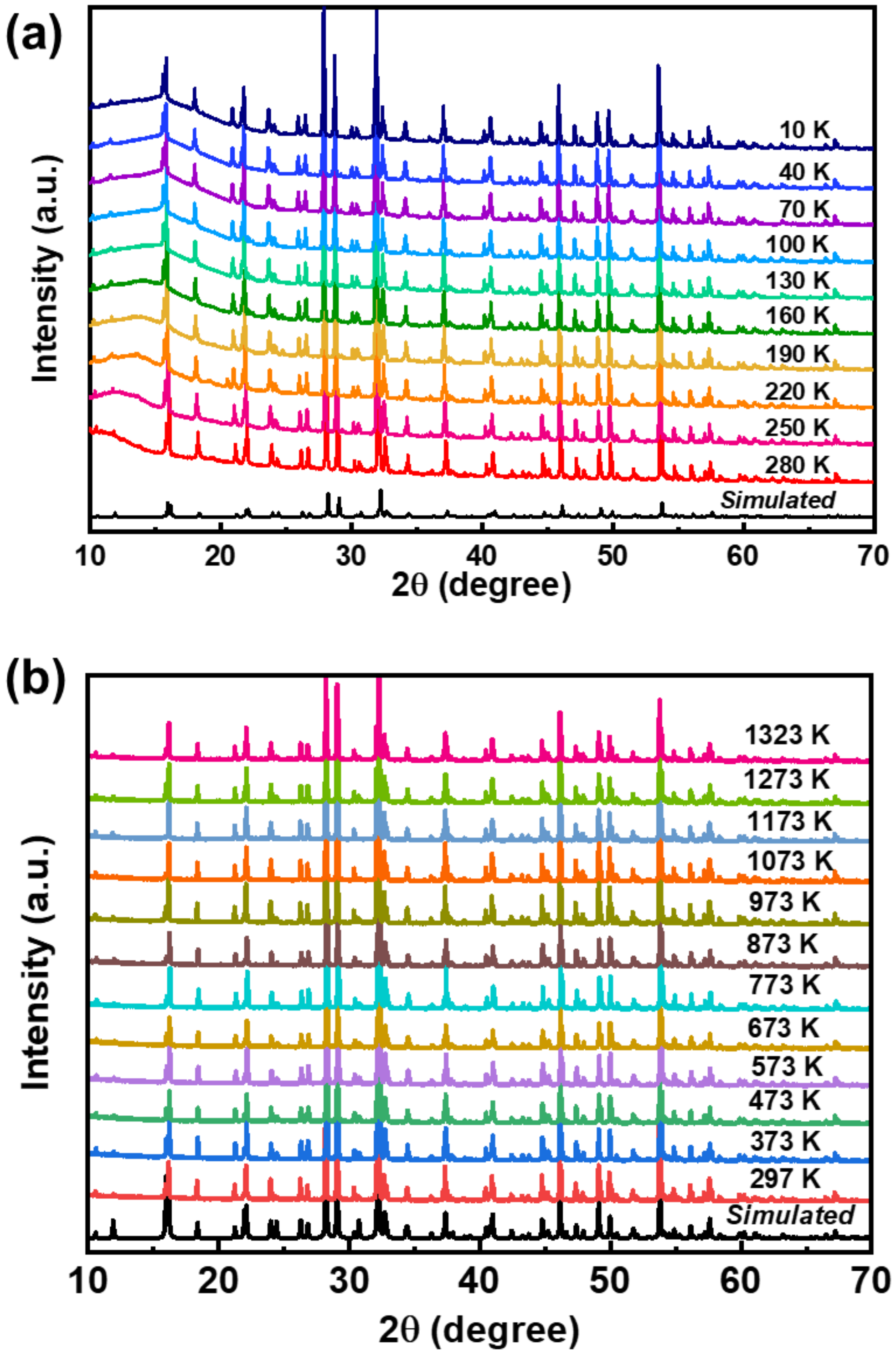

Supplementary Figure 4. *In-situ* variable-temperature powder XRD (a) and variable-temperature powder XRD (b) of the $LaMg_6Ga_6S_{16}$.

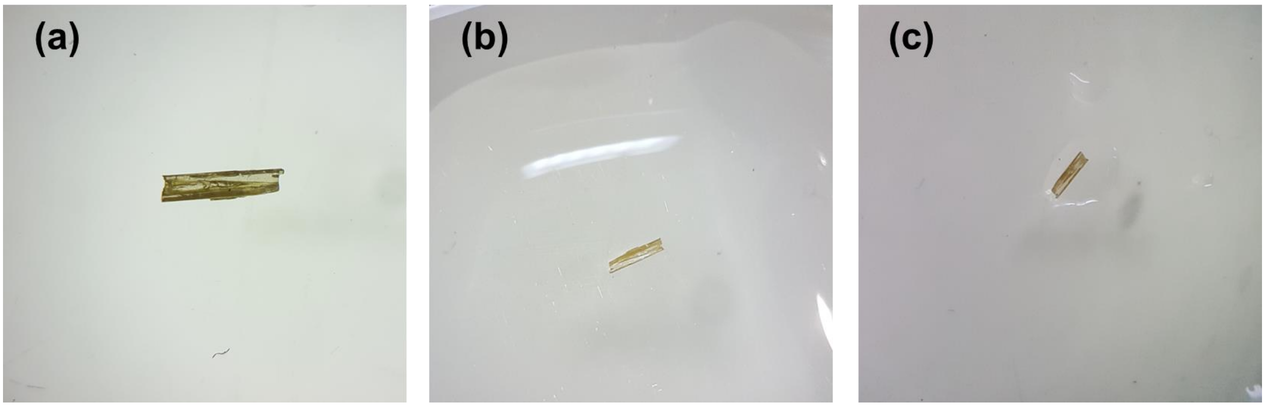

Supplementary Figure 5. The moisture stable $LaMg_6Ga_6S_{16}$: the crystal was soaked in the water for 7d (a) before soaking; (b) soaking in the water; (c) after soaking.

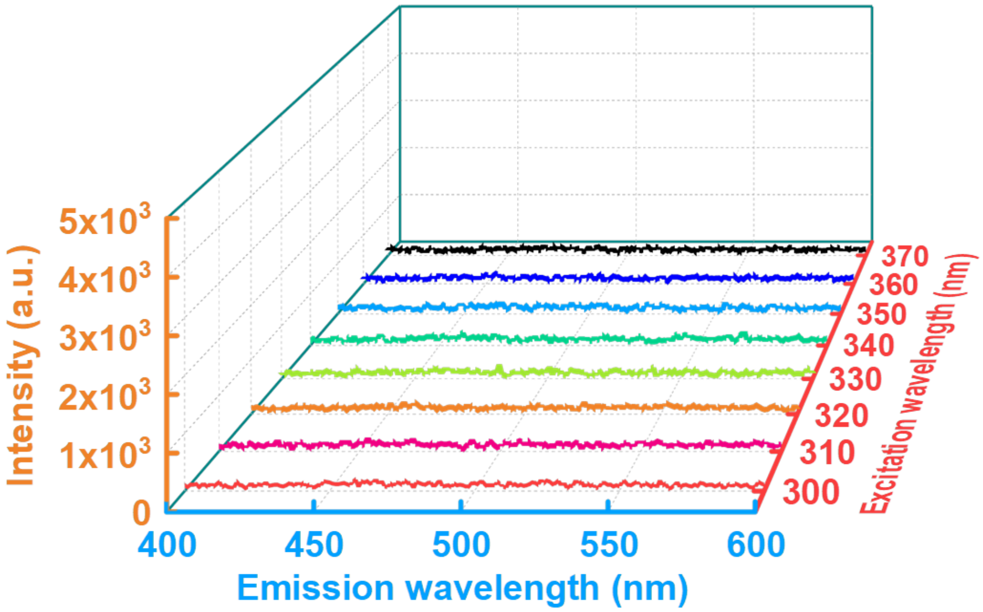

Supplementary Figure 6. Excitation-dependent PL spectra of $SrMg_6Ga_6S_{16}$ at room temperature.

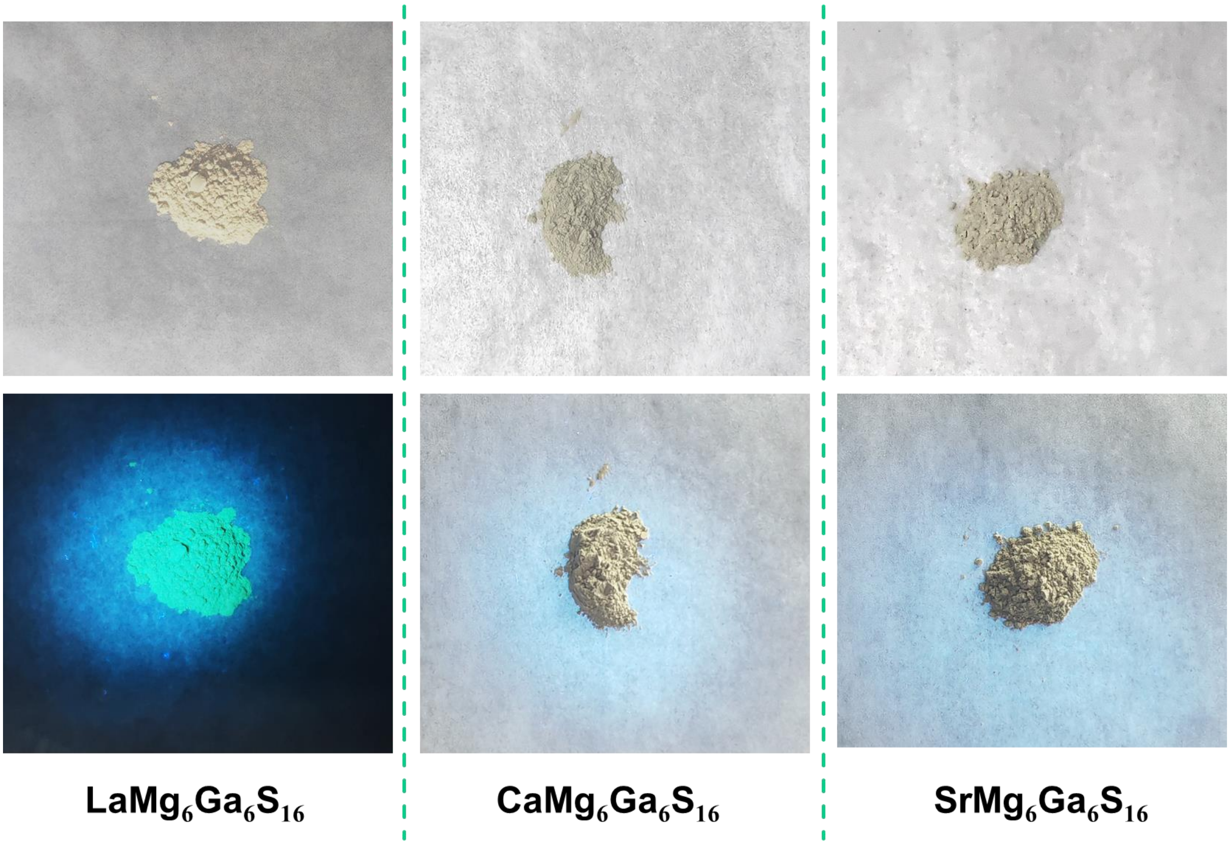

Supplementary Figure 7. Optical images of $LaMg_6Ga_6S_{16}$, $CaMg_6Ga_6S_{16}$, and $SrMg_6Ga_6S_{16}$ under UV irradiation at room temperature.

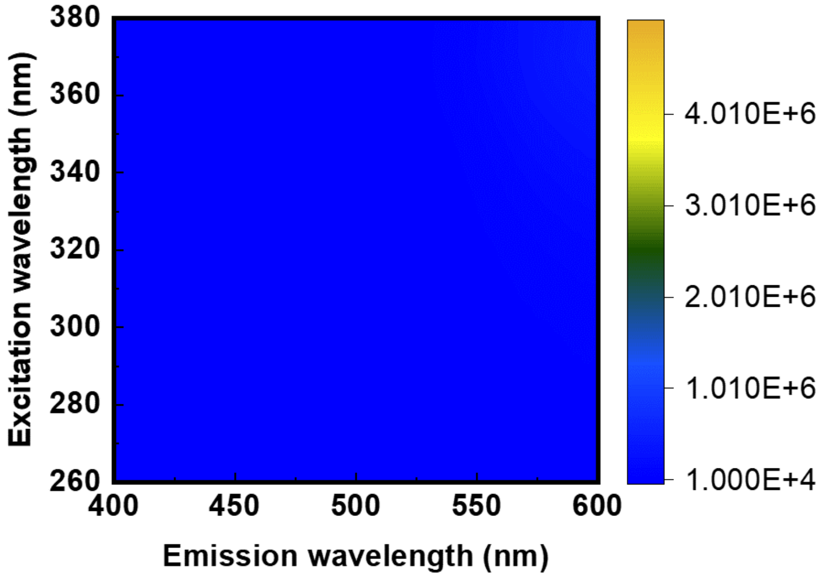

Supplementary Figure 8. Excitation-dependent PL spectra of La_2S_3 at the room temperature.

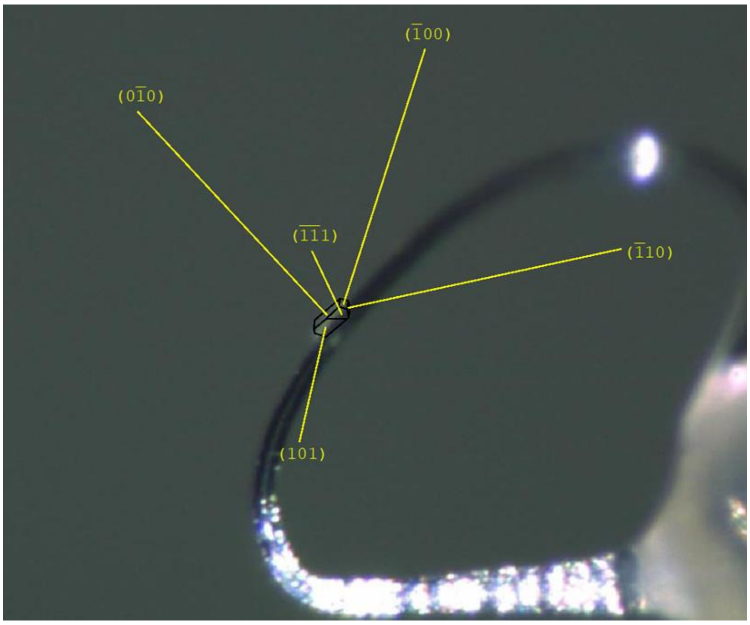

Supplementary Figure 9. The crystal orientation of the $LaMg_6Ga_6S_{16}$ was indexed by using a Bruker SMART APEX III.

Supplementary Table 1. Crystallographic Data and Refinement Details for $LaMg_6Ga_6S_{16}$.

Empirical formula	$LaMg_6Ga_6S_{16}$
Formula weight	3648.16
Temperature (K)	297(2)
Crystal system	Hexagonal
Space group	$P\bar{6}$
Z	1
a (Å)	16.7154(5)
c (Å)	7.4147(3)
V (Å^3)	1794.15(13)
D_c (g cm^{-3})	3.376
μ (mm^{-1})	9.949
F(000)	1713
Radiation	Mo-K_α (λ = 0.71073)
2θ range(°)	2.44 to 25.34
Reflections collected	9331
Indep. Reflns/ Rint	2340/0.0393
GOOF on F^2	1.184
R_1, wR_2 (I >2σ(I))[a]	0.0423, 0.1211
R_1, wR_2 (all data)	0.0495, 0.1283
largest diff. peak and hole (e·Å^{-3})	4.134, -2.213

[a]$R_1 = \Sigma||F_o|—|F_c||/\Sigma|F_o|$, [b]$wR_2 = \Sigma w(F_o^2—F_c^2)^2/\Sigma w(F_o^2)^2]^{1/2}$.

Supplementary Table 2. Atomic coordinates (× 10^4) and equivalent isotropic displacement parameters (Å^2 × 10^3) for $LaMg_6Ga_6S_{16}$. U_{eq} is defined as one-third of the trace of the orthogonalized U_{ij} tensor.

Atom	BVS[a]	*Wyckoff*	*x*	*y*	*z*	U_{eq}*(Å)*
La(1)	2.13	1*c*	3333	6667	10000	44(1)
La(2)	1.96	1*a*	0	0	10000	44(1)
La(3)	2.10	1*e*	6667	3333	10000	46(1)
Mg(1)	2.05	3*k*	4292(9)	1271(8)	5000	30(3)
Mg(2)	2.04	3*j*	4206(10)	1332(10)	10000	39(3)
Mg(3)	1.94	3*k*	2019(7)	2432(8)	5000	25(2)
Mg(4)	1.96	3*j*	2075(8)	2571(10)	10000	35(3)
Mg(5)	1.87	3*k*	3791(7)	4736(7)	5000	28(3)
Mg(6)	1.80	3*j*	3829(7)	4658(9)	10000	42(4)
Ga(1)	3.00	6*l*	5603(2)	7034(2)	7482(4)	26(1)
Ga(2)	2.97	6*l*	-318(2)	1872(2)	7468(4)	26(1)
Ga(3)	2.97	6*l*	4702(2)	3573(2)	7474(4)	25(1)
S(1)	2.05	6*l*	5050(4)	2450(5)	7545(7)	27(1)
S(2)	2.18	6*l*	-106(4)	3335(4)	7510(20)	27(1)
S(3)	1.95	6*l*	876(4)	1628(5)	7513(8)	27(1)
S(4)	1.98	6*l*	3264(4)	3371(4)	7510(20)	25(1)
S(5)	1.96	6*l*	4134(5)	5868(5)	7506(7)	27(1)
S(6)	1.93	3*k*	-1278(7)	1346(7)	5000	27(2)
S(7)	1.82	3*j*	5375(7)	4503(7)	10000	27(2)
S(8)	2.02	3*j*	5837(7)	7925(7)	10000	31(2)
S(9)	2.02	3*k*	5957(5)	7963(7)	5000	24(2)
S(10)	2.03	3*k*	5286(6)	4542(7)	5000	26(2)
S(11)	1.84	3*j*	-1222(7)	1237(7)	10000	33(2)

[a]Bond valence state was calculated using the empirical formula $V_i = \Sigma S_{ij} = \Sigma \exp[(r_0 - r_{ij})/0.37]$, where S_{ij} is the bond valence associated with bond lengths r_{ij} and r_0.[1, 2, 3, 4]

Supplementary Table 3. Selected distances (Å) and angles (degrees) for $LaMg_6Ga_6S_{16}$.

La(1)-S(5)#1	2.963(7)	Mg(2)-S(11)#8	2.834(18)
La(1)-S(5)	2.963(7)	Mg(3)-S(3)#15	2.522(10)
La(1)-S(5)#2	2.963(7)	Mg(3)-S(3)	2.522(10)
La(1)-S(5)#3	2.963(7)	Mg(3)-S(6)#8	2.587(15)
La(1)-S(5)#4	2.963(7)	Mg(3)-S(4)	2.643(15)
La(1)-S(5)#5	2.963(7)	Mg(3)-S(4)#15	2.643(15)
La(2)-S(3)	2.994(7)	Mg(3)-S(9)#3	2.705(14)
La(2)-S(3)#1	2.994(7)	Mg(4)-S(4)	2.548(16)
La(2)-S(3)#6	2.994(7)	Mg(4)-S(4)#1	2.548(16)
La(2)-S(3)#7	2.994(7)	Mg(4)-S(3)	2.597(11)
La(2)-S(3)#8	2.994(7)	Mg(4)-S(3)#1	2.597(11)
La(2)-S(3)#9	2.994(7)	Mg(4)-S(11)#8	2.635(17)
La(3)-S(1)#10	2.967(6)	Mg(4)-S(8)#3	2.650(17)
La(3)-S(1)#11	2.967(6)	Mg(5)-S(5)	2.505(10)
La(3)-S(1)#1	2.967(6)	Mg(5)-S(5)#15	2.505(10)
La(3)-S(1)	2.967(6)	Mg(5)-S(9)#3	2.607(14)
La(3)-S(1)#12	2.967(6)	Mg(5)-S(10)	2.675(16)
La(3)-S(1)#13	2.967(6)	Mg(5)-S(4)#15	2.727(15)
Mg(1)-S(10)#13	2.508(13)	Mg(5)-S(4)	2.727(15)
Mg(1)-S(2)#14	2.552(16)	Mg(6)-S(5)	2.596(12)
Mg(1)-S(2)#8	2.552(16)	Mg(6)-S(5)#1	2.596(12)
Mg(1)-S(1)	2.560(10)	Mg(6)-S(8)#3	2.597(18)
Mg(1)-S(1)#15	2.560(10)	Mg(6)-S(4)#1	2.627(16)
Mg(1)-S(6)#8	2.793(15)	Mg(6)-S(4)	2.627(16)
Mg(2)-S(1)#1	2.482(11)	Mg(6)-S(7)	2.723(18)
Mg(2)-S(1)	2.482(11)	Ga(1)-S(5)	2.245(6)
Mg(2)-S(2)#8	2.572(16)	Ga(1)-S(2)#5	2.278(6)
Mg(2)-S(2)#7	2.572(16)	Ga(1)-S(9)	2.287(7)
Mg(2)-S(7)#13	2.628(16)	Ga(1)-S(8)	2.297(7)
Ga(2)-S(3)	2.228(6)	S(1)#10-La(3)-S(1)#11	75.7(2)
Ga(2)-S(2)	2.290(6)	S(1)#10-La(3)-S(1)#1	86.30(16)
Ga(2)-S(6)	2.299(7)	S(1)#11-La(3)-S(1)#1	133.49(8)
Ga(2)-S(11)	2.309(7)	S(1)#10-La(3)-S(1)	133.49(7)
Ga(3)-S(1)	2.226(7)	S(10)#13-Mg(1)-S(2)#14	88.1(3)
Ga(3)-S(4)	2.253(6)	S(10)#13-Mg(1)-S(2)#8	88.1(3)
Ga(3)-S(10)	2.315(7)	S(2)#14-Mg(1)-S(2)#8	93.8(7)

Ga(3)-S(7)	2.333(6)	S(10)#13-Mg(1)-S(1)	95.6(4)
S(5)#1-La(1)-S(5)	77.2(3)	S(2)#14-Mg(1)-S(1)	176.2(5)
S(5)#1-La(1)-S(5)#2	85.18(19)	S(2)#8-Mg(1)-S(1)	85.5(3)
S(5)-La(1)-S(5)#2	134.00(9)	S(10)#13-Mg(1)-S(1)#15	95.6(4)
S(5)#1-La(1)-S(5)#3	134.00(9)	S(2)#14-Mg(1)-S(1)#15	85.5(3)
S(5)-La(1)-S(5)#3	85.18(19)	S(2)#8-Mg(1)-S(1)#15	176.2(5)
S(5)#2-La(1)-S(5)#3	134.00(9)	S(1)-Mg(1)-S(1)#15	94.9(5)
S(5)#1-La(1)-S(5)#4	85.18(19)	S(10)#13-Mg(1)-S(6)#8	162.5(6)
S(5)-La(1)-S(5)#4	134.00(9)	S(2)#14-Mg(1)-S(6)#8	80.0(3)
S(5)#2-La(1)-S(5)#4	85.17(19)	S(2)#8-Mg(1)-S(6)#8	80.0(3)
S(5)#3-La(1)-S(5)#4	77.2(3)	S(1)-Mg(1)-S(6)#8	96.2(3)
S(5)#1-La(1)-S(5)#5	134.00(9)	S(1)#15-Mg(1)-S(6)#8	96.2(3)
S(5)-La(1)-S(5)#5	85.18(19)	S(1)#1-Mg(2)-S(1)	94.3(6)
S(5)#2-La(1)-S(5)#5	77.2(3)	S(1)#1-Mg(2)-S(2)#8	174.2(6)
S(5)#3-La(1)-S(5)#5	85.17(19)	S(1)-Mg(2)-S(2)#8	86.7(3)
S(5)#4-La(1)-S(5)#5	134.00(9)	S(1)#1-Mg(2)-S(2)#7	86.7(3)
S(3)-La(2)-S(3)#1	76.0(2)	S(1)-Mg(2)-S(2)#7	174.2(6)
S(3)-La(2)-S(3)#6	86.04(18)	S(2)#8-Mg(2)-S(2)#7	91.6(7)
S(3)#1-La(2)-S(3)#6	133.60(8)	S(1)#1-Mg(2)-S(7)#13	88.8(4)
S(3)-La(2)-S(3)#7	133.60(8)	S(1)-Mg(2)-S(7)#13	88.8(4)
S(3)#1-La(2)-S(3)#7	86.04(18)	S(2)#8-Mg(2)-S(7)#13	85.5(4)
S(3)#6-La(2)-S(3)#7	133.60(8)	S(2)#7-Mg(2)-S(7)#13	85.5(4)
S(3)-La(2)-S(3)#8	86.05(18)	S(1)#1-Mg(2)-S(11)#8	103.2(4)
S(3)#1-La(2)-S(3)#8	133.60(8)	S(1)-Mg(2)-S(11)#8	103.2(4)
S(3)#6-La(2)-S(3)#8	86.04(18)	S(2)#8-Mg(2)-S(11)#8	82.1(4)
S(3)#7-La(2)-S(3)#8	76.0(2)	S(2)#7-Mg(2)-S(11)#8	82.1(4)
S(3)-La(2)-S(3)#9	133.60(8)	S(7)#13-Mg(2)-S(11)#8	162.1(6)
S(3)#1-La(2)-S(3)#9	86.05(18)	S(3)#15-Mg(3)-S(3)	95.2(4)
S(3)#6-La(2)-S(3)#9	76.0(2)	S(3)#15-Mg(3)-S(6)#8	94.4(4)
S(3)#7-La(2)-S(3)#9	86.04(18)	S(3)-Mg(3)-S(6)#8	94.4(4)
S(3)#8-La(2)-S(3)#9	133.60(8)	S(3)#15-Mg(3)-S(4)	176.4(5)
S(1)#11-La(3)-S(1)	86.30(16)	S(3)-Mg(3)-S(4)	87.6(3)
S(1)#1-La(3)-S(1)	75.7(2)	S(6)#8-Mg(3)-S(4)	87.6(3)
S(1)#10-La(3)-S(1)#12	86.30(16)	S(3)#15-Mg(3)-S(4)#15	87.6(3)
S(1)#11-La(3)-S(1)#12	133.49(8)	S(3)-Mg(3)-S(4)#15	176.4(5)
S(1)#1-La(3)-S(1)#12	86.30(16)	S(6)#8-Mg(3)-S(4)#15	87.6(3)
S(1)-La(3)-S(1)#12	133.49(8)	S(4)-Mg(3)-S(4)#15	89.5(6)
S(1)#10-La(3)-S(1)#13	133.49(8)	S(3)#15-Mg(3)-S(9)#3	98.6(4)

S(1)#11-La(3)-S(1)#13	86.30(16)	S(3)-Mg(3)-S(9)#3	98.6(4)
S(1)#1-La(3)-S(1)#13	133.49(8)	S(6)#8-Mg(3)-S(9)#3	160.6(5)
S(1)-La(3)-S(1)#13	86.30(16)	S(4)-Mg(3)-S(9)#3	78.8(3)
S(1)#12-La(3)-S(1)#13	75.7(2)	S(11)#8-Mg(4)-S(8)#3	167.4(6)
S(4)-Mg(4)-S(4)#1	92.9(7)	S(5)#1-Mg(6)-S(8)#3	90.0(4)
S(4)-Mg(4)-S(3)	88.1(3)	S(5)-Mg(6)-S(4)#1	171.6(6)
S(4)#1-Mg(4)-S(3)	174.9(7)	S(5)#1-Mg(6)-S(4)#1	89.3(3)
S(4)-Mg(4)-S(3)#1	174.9(7)	S(8)#3-Mg(6)-S(4)#1	81.5(4)
S(4)#1-Mg(4)-S(3)#1	88.1(3)	S(5)-Mg(6)-S(4)	89.3(3)
S(3)-Mg(4)-S(3)#1	90.5(5)	S(5)#1-Mg(6)-S(4)	171.5(6)
S(4)-Mg(4)-S(11)#8	89.4(4)	S(8)#3-Mg(6)-S(4)	81.5(4)
S(4)#1-Mg(4)-S(11)#8	89.4(4)	S(4)#1-Mg(6)-S(4)	89.3(7)
S(3)-Mg(4)-S(11)#8	85.7(4)	S(5)-Mg(6)-S(7)	104.4(4)
S(3)#1-Mg(4)-S(11)#8	85.7(4)	S(5)#1-Mg(6)-S(7)	104.4(4)
S(4)-Mg(4)-S(8)#3	82.0(4)	S(8)#3-Mg(6)-S(7)	159.3(7)
S(4)#1-Mg(4)-S(8)#3	82.0(4)	S(4)#1-Mg(6)-S(7)	83.8(4)
S(3)-Mg(4)-S(8)#3	103.1(4)	S(4)-Mg(6)-S(7)	83.8(4)
S(3)#1-Mg(4)-S(8)#3	103.1(4)	S(5)-Ga(1)-S(2)#5	113.7(2)
S(5)-Mg(5)-S(5)#15	95.8(5)	S(5)-Ga(1)-S(9)	113.3(3)
S(5)-Mg(5)-S(9)#3	96.6(4)	S(2)#5-Ga(1)-S(9)	105.2(4)
S(5)#15-Mg(5)-S(9)#3	96.6(4)	S(5)-Ga(1)-S(8)	108.3(3)
S(5)-Mg(5)-S(10)	102.5(4)	S(2)#5-Ga(1)-S(8)	108.0(5)
S(5)#15-Mg(5)-S(10)	102.5(4)	S(9)-Ga(1)-S(8)	108.1(3)
S(9)#3-Mg(5)-S(10)	151.3(6)	S(3)-Ga(2)-S(2)	121.4(3)
S(5)-Mg(5)-S(4)#15	173.9(5)	S(3)-Ga(2)-S(6)	117.4(3)
S(5)#15-Mg(5)-S(4)#15	89.0(3)	S(2)-Ga(2)-S(6)	97.2(4)
S(9)#3-Mg(5)-S(4)#15	79.0(3)	S(3)-Ga(2)-S(11)	110.1(3)
S(10)-Mg(5)-S(4)#15	80.2(4)	S(2)-Ga(2)-S(11)	101.3(5)
S(5)-Mg(5)-S(4)	89.0(3)	S(6)-Ga(2)-S(11)	107.5(3)
S(5)#15-Mg(5)-S(4)	173.9(5)	S(1)-Ga(3)-S(4)	125.6(3)
S(9)#3-Mg(5)-S(4)	79.0(3)	S(1)-Ga(3)-S(10)	114.3(3)
S(10)-Mg(5)-S(4)	80.2(4)	S(4)-Ga(3)-S(10)	99.2(4)
S(4)#15-Mg(5)-S(4)	86.0(7)	S(1)-Ga(3)-S(7)	107.5(3)
S(5)-Mg(6)-S(5)#1	90.9(5)	S(4)-Ga(3)-S(7)	102.3(5)
S(5)-Mg(6)-S(8)#3	90.0(4)	S(10)-Ga(3)-S(7)	106.0(3)

Symmetry transformations used to generate equivalent atoms:

#1 x,y,-z+2 #2 -y+1,x-y+1,-z+2 #3 -x+y,-x+1,z

#4 -x+y,-x+1,-z+2 #5 -y+1,x-y+1,z #6 -y,x-y,z

#7 -x+y,-x,-z+2   #8 -x+y,-x,z   #9 -y,x-y,-z+2
#10 -x+y+1,-x+1,-z+2   #11 -x+y+1,-x+1,z   #12 -y+1,x-y,-z+2
#13 -y+1,x-y,z   #14 -x+y,-x,-z+1   #15 x,y,-z+1

Supplementary Table 4 The space groups and the flexibility indices (*F*) of GaS_4 and MgS_6 groups in $AgGaS_2$, $LiGaS_2$, α-$BaGa_4S_7$, $CaMg_6Ga_6S_{16}$, and $LaMg_6Ga_6S_{16}$.

Compounds	Space groups	GaS_4/MgS_6 groups	*F*
$AgGaS_2$	*I*-42*d*	GaS_4	0.212
$LiGaS_2$	*Pna*2$_1$	GaS_4	0.220
α-$BaGa_4S_7$	*Pmn*2$_1$	GaS_4	0.216
$CaMg_6Ga_6S_{16}$	*P*-6	GaS_4	0.220-0.224
		MgS_6	0.140-0.149
$LaMg_6Ga_6S_{16}$	*P*-6	GaS_4	0.220-0.222
		MgS_6	0.138-0.149
		LaS_6	0.101-0.112

As the SHG response of a material is relevant to the polarizability of the NLO-active anionic groups, the induced polarizability of [MgS_6] and [GaS_4] groups were quantified based on the calculation of empirical "flexibility index" *F* by the bond valence method.[4] As displayed in Table 6, the magnitudes of the induced dipole moment of [GaS_4] groups are comparable with other chalcogenides with large SHG responses, *e.g.,* $AgGaS_2$, $LiGaS_2$, $BaGa_4S_7$, and $CaMg_6Ga_6S_{16}$. Meanwhile, the [MgS_6] and [LaS_6]octahedra make also non-negligible contributions to the induced dipole moments in $LaMg_6Ga_6S_{16}$. These illustrate that these NLO-active groups will induce large polarizations under the external field perturbation, thereby making a positive contribution to the SHG response.

Supplementary Table 5. NLO properties in $LaMg_6Ga_6S_{16}$ and other reported chalcogenides.

Compound	Space group	d_{ij} (× AGS, pm/V)	LIDT(× AGS)	PM/NPM	Δ*n*@1064 nm	Band gap (eV)
Ga_2S_3[5]	*Cc*	0.3	30	NPM	0.025@1064 nm	2.80
$AgGaS_2$[6]	*I*-42*m*	d_{36} = 13.4	*1*	PM	0.044@1064 nm	2.56
α-$BaGa_4S_7$[7]	*Pmn*2$_1$	0.9	3	PM	0.111@1064 nm	3.54
$PbGa_4S_7$[8]	*Pc*	1.2		PM		3.08
$SnGa_4S_7$[9]	*Pc*	1.3	19	PM		3.10
$MgGa_2Se_4$[10]	*I*-4	0.9	3	PM	0.048@546 nm	2.96
$AgGaGeS_4$[11]	*Fdd*2	0.9	2.5	PM		2.78
$Na_2ZnGe_2S_6$[12]	*Cc*	0.9	6	PM		3.25
Na_2BaGeS_4[13]	*R*3*c*	0.3	8	PM	0.037@1064 nm	3.70
Na_2BaSnS_4[13]	*I*-42*d*	0.5	5	PM	0.070@1064 nm	3.27
$Li_2Ga_2GeS_6$[14]	*Fdd*2	0.8		PM		2.95
$KYGeS_4$[15]	*P*2$_1$	1.0		PM	0.120@1064 nm	3.15
$RbMn_3Ga_5S_{11}$[16]	*Pc*	0.7	31.1	PM	0.017@2050 nm	3.27
$CsMn_3Ga_5S_{11}$[16]	*Pc*	0.8	29.1	PM	0.016@2050 nm	3.23
$Sr_5ZnGa_6S_{15}$[17]	*Ama*2	1.2	16	PM	0.047@2090 nm	3.15
$Ba_6Zn_7Ga_2S_{16}$[18]	*R*3	0.5	28	PM		3.50
$Ba_2Ga_8GeS_{16}$[19]	*P*6$_3$*mc*	1.0	22	PM		3.00
$BaGa_2GeS_6$[20]	*R*3	1.0				3.23
$LaMg_6Ga_6S_{16}$	*P*-6	0.8	5	PM	0.041@1064 nm	3.50

Supplementary Table 6. The space groups and SHG coefficients of $LaMg_6Ga_6S_{16}$ and $AeMg_6Ga_6S_{16}$ (Ae = Ca, Sr, Ba).

Compounds	Space groups	d_{ij} (pm/V)	
$LaMg_6Ga_6S_{16}$	*P*-6	d_{11} = 12.27	d_{22} = 4.00
$CaMg_6Ga_6S_{16}$.	*P*-6	d_{11} = 9.9	d_{22} = 3.82
$SrMg_6Ga_6S_{16}$.	*P*-6	d_{11} = 10.1	d_{22} = 3.83
$BaMg_6Ga_6S_{16}$.	*P*-6	d_{11} = 9.9	d_{22} = 3.87

Supplementary References

1. Wang, J.; Cheng, Y.; Wu, H.; Hu, Z.; Wang, J.; Wu, Y.; Yu, H. $Sr_3[SnOSe_3][CO_3]$: A Heteroanionic Nonlinear Optical Material Containing Planar pi-conjugated $[CO_3]$ and Heteroleptic $[SnOSe_3]$ Anionic Groups. *Angew. Chem. Int. Ed.* **61**, e202201616 (2022).
2. Brown I, Altermatt D. Bond-valence parameters obtained from a systematic analysis of the Inorganic Crystal Structure Database. *Acta Crystallogr. A* **41**, 244-247 (2010).
3. Brown I. Recent Developments in the Methods and Applications of the Bond Valence Model. *Chem. Rev.* **109**, 6858-6919 (2009).
4. Jiang X, et al. The role of dipole moment in determining the nonlinear optical behavior of materials: ab initio studies on quaternary molybdenum tellurite crystals. *J. Mater. Chem. C* **2**, 530-537 (2014).
5. Zhang M, Jiang X, Zhou L, Guo G. Two phases of Ga_2S_3: promising infrared second-order nonlinear optical materials with very high laser induced damage thresholds. *J. Mater. Chem. C* **1**, 4754-4760 (2013).
6. Okorogu A, et al. Tunable middle infrared downconversion in GaSe and $AgGaS_2$. *Opt. Commun.* **155**, 307-312 (1998).
7. Lin X, Zhang G, Ye N. Growth and Characterization of $BaGa_4S_7$: A New Crystal for Mid-IR Nonlinear Optics. *Cryst. Growth. Des.* **9**, 1186-1189 (2009).
8. Li X, Kang L, Li C, Lin Z, Yao J, Wu Y. $PbGa_4S_7$: a wide-gap nonlinear optical material. *J. Mater. Chem. C* **3**, 3060-3067 (2015).
9. Luo Z, et al. SHG Materials $SnGa_4Q_7$ (Q = S, Se) Appearing with Large Conversion Efficiencies, High Damage Thresholds, and Wide Transparencies in the Mid-Infrared Region. *Chem. Mater.* **26**, 2743-2749 (2014).
10. Wang P, et al. The Combination of Structure Prediction and Experiment for the Exploration of Alkali-Earth Metal-Contained Chalcopyrite-Like IR Nonlinear Optical Material. *Adv. Sci.* **9**, 2106120 (2022).
11. Petrov V, Badikov V, Shevyrdyaeva G, Panyutin V, Chizhikov V. Phase-matching properties and optical parametric amplification in single crystals of $AgGaGeS_4$. *Opt. Mater*. **26**, 217-222 (2004).
12. Li G, Wu K, Liu Q, Yang Z, Pan S. $Na_2ZnGe_2S_6$: A New Infrared Nonlinear Optical Material with Good Balance between Large Second-Harmonic Generation Response and High Laser Damage Threshold. *J. Am. Chem. Soc.* **138**, 7422-7428 (2016).
13. Wu K, Yang Z, Pan S. Na_2BaMQ_4 (M=Ge, Sn; Q=S, Se): Infrared Nonlinear Optical Materials with Excellent Performances and that Undergo Structural Transformations. *Angew. Chem. Int. Ed.* **55**, 6713-6715 (2016).
14. Kim Y, et al. Characterization of New Infrared Nonlinear Optical Material with High Laser Damage Threshold, $Li_2Ga_2GeS_6$. *Chem. Mater.* **20**, 6048-6052 (2008).
15. Mei D, et al. Breaking through the “3.0 eV wall” of energy band gap in mid-infrared nonlinear optical rare earth chalcogenides by charge-transfer engineering. *Mater. Horiz.* **8**, 2330-2334 (2021).
16. Chen M, Zhou S, Wei W, Wu X, Lin H, Zhu Q. Phase Matchability Transformation in the Infrared Nonlinear Optical Materials with Diamond-Like Frameworks. *Adv. Opt. Mater.* **10**, 2102123 (2022).
17. Lin H, et al. $Sr_5ZnGa_6S_{15}$: a new quaternary non-centrosymmetric semiconductor with a 3D framework structure displaying excellent nonlinear optical performance. *Inorg. Chem. Front.* **5**, 1458-1462 (2018).
18. Li Y, Liu P, Wu L. $Ba_6Zn_7Ga_2S_{16}$: A Wide Band Gap Sulfide with Phase-Matchable Infrared NLO Properties. *Chem. Mater.* **29**, 5259-5266 (2017).
19. Liu B, et al. Syntheses, Structures, and Nonlinear-Optical Properties of Metal Sulfides $Ba_2Ga_8MS_{16}$ (M = Si, Ge). *Inorg. Chem.* **54**, 976-981 (2015).

20. Yin W, et al. $BaGa_2MQ_6$ (M = Si, Ge; Q = S, Se): a new series of promising IR nonlinear optical materials. *Dalton. Trans.* **41**, 5653-5661 (2012).
